# Opinion mining for national security: techniques, domain applications, challenges and research opportunities

**DOI:** 10.1186/s40537-021-00536-5

**Published:** 2021-12-04

**Authors:** Noor Afiza Mat Razali, Nur Atiqah Malizan, Nor Asiakin Hasbullah, Muslihah Wook, Norulzahrah Mohd Zainuddin, Khairul Khalil Ishak, Suzaimah Ramli, Sazali Sukardi

**Affiliations:** 1 National Defence University of Malaysia, Kuala Lumpur, Malaysia; 2 Management and Science University, Selangor, Malaysia; 3 CyberSecurity Malaysia, Selangor, Malaysia

**Keywords:** Opinion mining, Sentiment analysis, National security, Machine learning, Lexicon-based approach, Kansei approach

## Abstract

**Background:**

Opinion mining, or sentiment analysis, is a field in Natural Language Processing (NLP). It extracts people’s thoughts, including assessments, attitudes, and emotions toward individuals, topics, and events. The task is technically challenging but incredibly useful. With the explosive growth of the digital platform in cyberspace, such as blogs and social networks, individuals and organisations are increasingly utilising public opinion for their decision-making. In recent years, significant research concerning mining people’s sentiments based on text in cyberspace using opinion mining has been explored. Researchers have applied numerous opinions mining techniques, including machine learning and lexicon-based approach to analyse and classify people’s sentiments based on a text and discuss the existing gap. Thus, it creates a research opportunity for other researchers to investigate and propose improved methods and new domain applications to fill the gap.

**Methods:**

In this paper, a structured literature review has been done by considering 122 articles to examine all relevant research accomplished in the field of opinion mining application and the suggested Kansei approach to solve the challenges that occur in mining sentiments based on text in cyberspace. Five different platforms database were systematically searched between 2015 and 2021: ACM (Association for Computing Machinery), IEEE (Advancing Technology for Humanity), SCIENCE DIRECT, SpringerLink, and SCOPUS.

**Results:**

This study analyses various techniques of opinion mining as well as the Kansei approach that will help to enhance techniques in mining people’s sentiment and emotion in cyberspace. Most of the study addressed methods including machine learning, lexicon-based approach, hybrid approach, and Kansei approach in mining the sentiment and emotion based on text. The possible societal impacts of the current opinion mining technique, including machine learning and the Kansei approach, along with major trends and challenges, are highlighted.

**Conclusion:**

Various applications of opinion mining techniques in mining people’s sentiment and emotion according to the objective of the research, used method, dataset, summarized in this study. This study serves as a theoretical analysis of the opinion mining method complemented by the Kansei approach in classifying people’s sentiments based on text in cyberspace. Kansei approach can measure people’s impressions using artefacts based on senses including sight, feeling and cognition reported precise results for the assessment of human emotion. Therefore, this research suggests that the Kansei approach should be a complementary factor including in the development of a dictionary focusing on emotion in the national security domain. Also, this theoretical analysis will act as a reference to researchers regarding the Kansei approach as one of the techniques to improve hybrid approaches in opinion mining.

## Introduction

Nowadays, cyberspace is consistently loaded with several applications and digital media where people with various backgrounds and expertise share their thoughts and opinions on numerous topics/events. Usually, the information shared by people is textual form-based [1]. Sharing can be made using any digital media application such as online news, blogs, and social media. Therefore, countless blogs, social media platforms, forums, news reports, e-commerce websites, and other online resources allow people to express opinions. Such information can be utilised to understand public and consumer opinions regarding product preferences, political movements, social events, marketing campaigns, company strategies, and monitoring reputations. People are unaware that the opinions they express have a negative impact on national security. A negative opinion can cause chaos and disputes among a community, which creates opposing views for people of other countries, thereby threatening a state’s national security [2].

To address this issue, communities of researchers and academicians have been rigorously working on sentiment analysis for the last decade and a half. Sentiment analysis (SA) is a computational assessment of the sentiments, opinions and emotions conveyed in texts and aimed at a certain entity [3]. Sentiment analysis (also called review mining, opinion mining, attitude analysis or appraisal extraction) is the task of detecting, extracting and classifying opinions, sentiments and attitudes concerning different topics, as expressed in textual input [4].

Opinion mining or sentiment analysis helps in achieving various goals such as observing public mood regarding political movements [5], customer satisfaction measurement [6], movie sales prediction [7], etc. However, the existing opinion mining method alone, which includes machine learning and lexicon-based approach, cannot effectively help in analysing and classifying people’s sentiments and emotions in cyberspace according to the national security domain because some opinion mining methods only focus on existing domains such as business and education. This paper suggests that the Kansei approach can be a complementary factor in mining and classifying people’s sentiment in other domains, such as the national security domain, by analysing suitable references for this approach.

The Kansei method can apply conventional techniques, such as consumer surveys and expert interviews, to understand people’s reactions towards a certain entity or event with the use of artefacts [8]. Kansei Engineering is one of the methods based on the Kansei approach, which has been employed in diverse research for emotional design. Kansei Engineering (KE) is capable of measuring people’s feelings and emotional states. These emotional and sensory outcomes are then translated into perceptual design elements of the product or artefact [9]. Typically, Kansei Words has proven to be excellent in describing affective needs and mapping relationships between Kansei words and design elements to achieve customers’ emotional satisfaction on product specifications. Nowadays, the Kansei approach can be used in different research areas such as education and information technology since the research method of KE had an influential effect on the relationship between the response of emotions and the attributes of any entity. Researchers are using this method in the information technology domain for analysing design elements for online websites. Therefore, this research explores the possible utilisation of KE in combination with other opinion mining methods to analyse emotions from the text.

This paper is structured as follows: Sect. “Introduction” provides a brief introduction on opinion mining and the Kansei approach and their functionality and application in mining people’s sentiments in cyberspace. Section “Method’ presents the method/research methodology employed in this paper with some explanation. Then, Sect. “Result” stated the result of the reviewed article, and Sect. “Discussion” explained and discussed the context of the result in depth. Section “Discussion” also discuss the finding by highlighting the functionalities of sentiment analysis/opinion mining and the Kansei approach as the new mechanism for mining people’s sentiment and emotions in the national security domain. Also, it presents the challenges of applying machine learning, the lexicon-based approach and the Kansei method for opinion mining based on text in cyberspace. Section “Future research directions of opinion mining for national security” discusses future research utilising the hybrid approach of machine learning, the lexicon-based approach and the Kansei approach for opinion mining in the national security domain. Section “Limitation” gives out the limitation of our research. Section “Conclusion” summarises the work, as well as the conclusion.

## Method

To observe the related literature on opinion mining/sentiment analysis and the Kansei approach in mining sentiments based on text in cyberspace, we conducted a systematic literature review of the relevant literature. The following research questions are our focus area on this paper:

1. How can opinion mining techniques and the Kansei approach enhance the methods of mining people’s sentiments and emotions in cyberspace?
2. What are the most relevant sectors that benefit from opinion mining which includes the Kansei approach?
3. What are the techniques used for opinion mining in various domain applications?
4. What are the challenges and future scope of research for opinion mining techniques that include the Kansei approach?

**Fig. 1:**
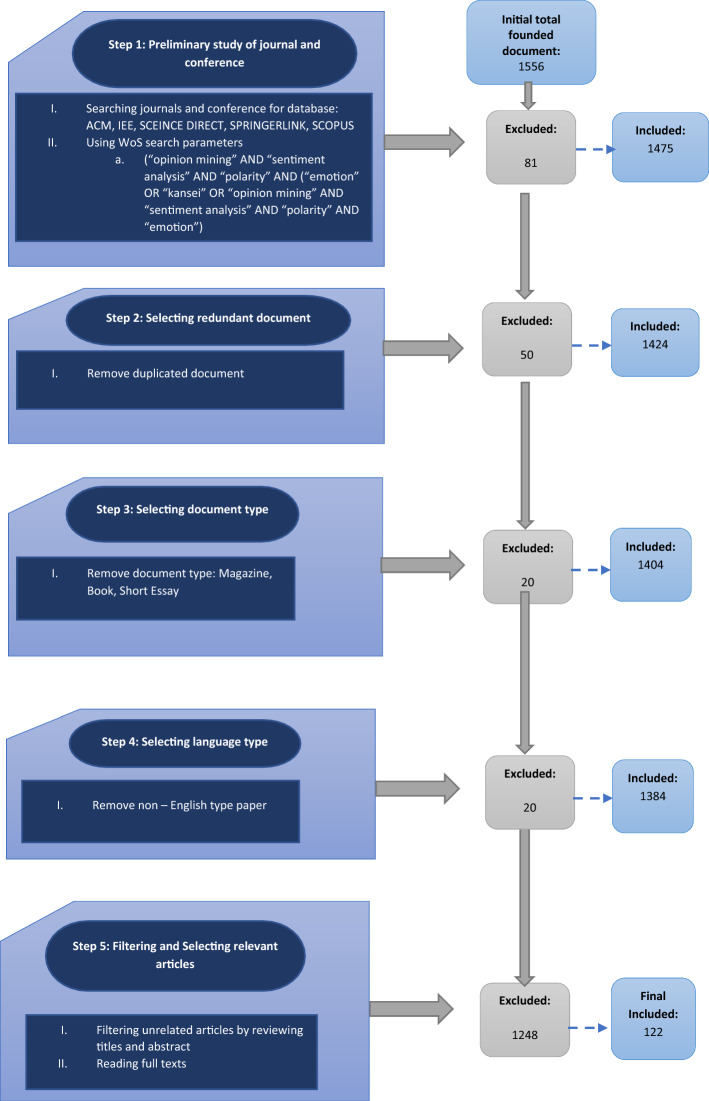
Research methodology

To answer the research questions above, we conducted the SLR by following the reference guidelines for performing systematic literature reviews in software engineering published by Kitchenham and Charters in 2007. A search has been conducted on five platforms: the ACM (Association for Computing Machinery), IEEE (Advancing Technology for Humanity), SCIENCE DIRECT, SpringerLink, and SCOPUS. Figure 1 presents the research methodology employed to find related articles.

Several keywords were selected to be used in this research, such as: “opinion mining,” “sentiment analysis,” “polarity,” “emotion,” “Kansei,” and “opinion mining.” The Web of Science operators such as ‘OR’ and ‘AND had been used in combination with the selected keyword for searching the particular publication. Based on the search platform, this research runs the searching by the keywords, title, or abstract.

Then, the result from the search was filtered through the inclusion or exclusion criteria. The research must follow the inclusion criteria, such as the publication year of the papers must be between 2015 and 2021, and the publication must write in English. The publication must be the focus on the opinion mining techniques based on text in cyberspace. Variety type of discipline was placed on the paper such as computer science, business, psychology, and medicine. Publication in the type of books, posters, and literature review was disregarded.

As the selection result, an initial set total of 1556 research documents was identified. The identified document was reduced to 1475 documents from the preliminary keyword search on the selected platforms. Then, the duplicated document was removed and gave out remaining a total of 1324 documents. The remaining 1324 documents have been checked and read based on the inclusion or exclusion criteria. After that process, a total of 1428 was excluded. The final of 122 relevant papers was included in this research, which is based on the evaluation on reading the full text of the papers. The subsequent section of the literature review involved the analysis of the remaining 122 articles.

## Result

**Fig. 2:**
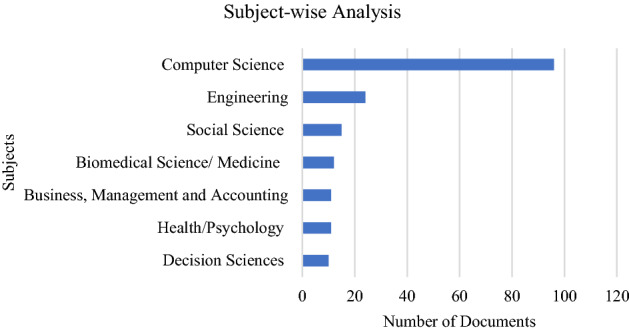
Subject-wise Analysis

In this paper, we study numerous subjects with 122 papers in total. We outline the descriptive statistics from the reviewed article, such as subject-wise analysis, year-wise analysis, and country-wise analysis. The chart in Fig. 2 shows the subject-wise classification; it reveals that Computer Science and Engineering are the major areas in which related research has been published. Social Sciences, Biomedical Science (Medicine), Health, Psychology, Business, Management, and Accounting and Decision Sciences have also observed an increase in the number of research publications on opinion mining/sentiment analysis and the Kansei approach for mining people’s sentiments in cyberspace.

**Fig. 3:**
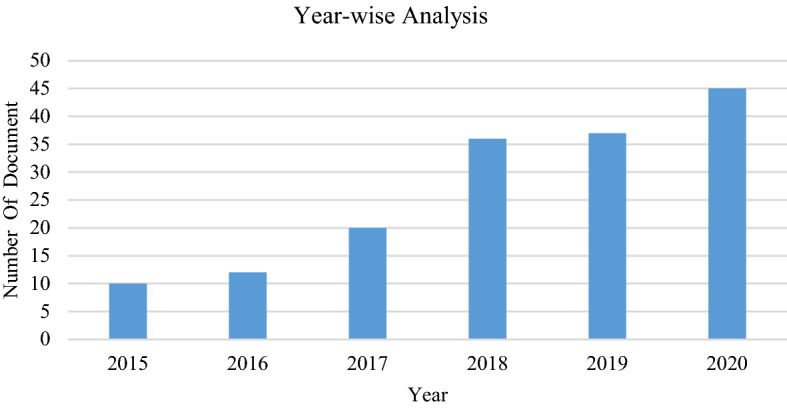
Year-wise Analysis

Based on the year-wise analysis, the significant research in opinion mining for analysing sentiments in cyberspace began from 2015 onwards. We can observe a substantial growth in the number of publications from 2015 to 2018. In 2020, an exponential increase can be seen with more papers published than in 2018, indicating a growing trend in this research area, as shown in Fig. 3. If we take a closer look at the research, many studies also concentrate on mining sentiments in cyberspace. It indicates that opinion mining is also being explored at a considerably faster rate across multiple industries, partially due to its growing use in various applications.

**Fig. 4:**
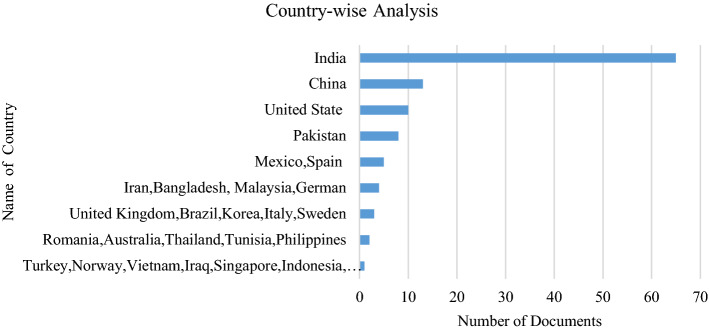
Country-wise analysis

Figure 4 illustrates the country-wise analysis; it presents the current trend regarding the location where India has the maximum amount of research published for opinion mining or sentiment analysis. However, United Stated (US) is also going forward and increasingly making contributions to the research. It shows that research on opinion mining has the potential to move further in enhancing the detection of people’s opinions in various domains. Asian nations and European nations such as Malaysia, Vietnam, South Korea, the United Kingdom (UK), and Italy also significantly contribute to this research area.

## Discussion

### Opinion mining overview

Sentiment analysis, also known as opinion mining, has been used to extract and interpret public sentiments and opinions for over a half-century by research communities, academics, government, and service industries. The role of opinion mining is both technically demanding and extremely realistic [10].

According to Liu [11], opinion mining/sentiment analysis is known as the computational study of people’s views, appraisals, attitudes and emotions toward individuals, people, problems, events, subjects, and their attributes. It is also the study of people’s opinions based on the sentiments, attitudes, or emotions expressed in a product [12].

‘A thought, opinion, or concept based on a feeling about a situation’ is the definition of the term “sentiment” according to the Cambridge dictionary [13]. Opinion mining involves the process of drawing opinions and categorising them according to their polarity, whether they are positive or negative or other emotions. They can be employed for different levels such as document-level sentiment analysis, sentence-level sentiment analysis, and feature or aspect-level sentiment analysis.

Opinion mining has been a research interest since the early twenty-first century. In 2003, Dave et al. [14] discussed opinion mining and proposed a model for document polarity classification (either recommended or not recommended) based on feedback analysis towards certain entities. From that research onwards, other researchers became interested in applying opinion mining in their text mining studies. It then became new extensive research in the following years. In 2004, Hu and Liu [15] had investigated the mining approach to summarise product reviews by identifying opinion sentences in each review and deciding whether each opinion sentence is positive or negative. In 2008, Abbasi et al. conducted research on sentiment analysis techniques and their applications [16, 17]. In 2009, Tang et al. [18] discussed document sentiment classification and opinion extraction and experimented with classifying web review opinions for consumer product analysis. In 2010, Chen and Zimbra [19] assessed the opinions of various business constituents regarding the company by employing an analysis framework that applied automatic topic and sentiment extraction methods to various online discussions. Based on the review of selected articles, this research found that between 2016 until today, opinion mining-related research is still an interesting subject area for researchers.

### Classification in opinion mining

There are various classification techniques that exist for sentiment or opinion mining. In classification, content polarity has been identified as a suitable approach to analyse people’s opinions interpreted in text. Usually, three classes are used for classification: positive, negative and neutral. According to the literature, most researchers have classified their sentiments as positive, negative and neutral. Singh et al. [20] and Akila et al. [21] had concluded in their findings that positive, negative and neutral opinions toward their entities are adequate. The classification algorithms used for sentiment analysis depend on the method employed, such as the supervised or unsupervised method.

### Techniques in performing opinion mining

To conduct opinion mining, researchers have recently applied various methods in the classification of opinions based on textual data. The supervised and unsupervised methods have been used as the classification algorithms. In the basic process of opinion mining, there are two well-known approaches. The unsupervised lexicon-based approach is one approach in which the process is guided by rules and heuristics derived from linguistic knowledge. Another approach is the supervised machine learning approach, where algorithms retrieve inherent information from existing labelled data in order to classify newer, unlabelled data [22].

**Fig. 5:**
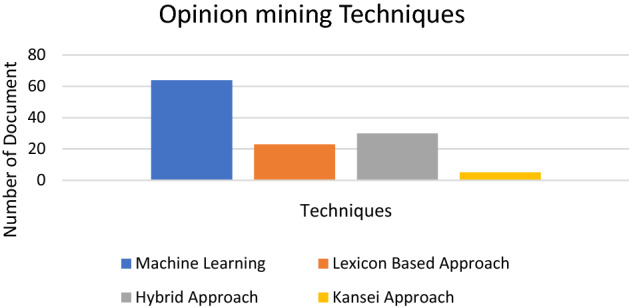
Opinion mining techniques chart

Followed by the research question on “What are the techniques used for opinion mining in various domain applications.” Based on the papers reviewed, all had shown the use of either the machine learning techniques, lexicon-based approach, or a mixture of both methods when executing sentiment analysis. The results reveal that opinion mining or sentiment analysis has been conducted in 64 papers using machine learning techniques, while 23 of the reviewed papers applied the lexicon-based approach and 30 papers presented a hybrid approach by combining both methods. Figure 5 displays a chart that contains the number of review papers according to the type of opinion mining technique. The following chart displays the number of review papers according to the type of opinion mining technique. Other techniques were also discussed in these papers, such as the Kansei approach. Five related papers have employed the Kansei approach for mining people’s opinions and emotions.

### Machine learning

The machine learning method is divided into three approaches: supervised learning, unsupervised learning and semi-supervised learning. Supervised learning uses labelled data that facilitate algorithms to learn and predict the sentiment of the text. Usually, to classify the opinion or sentiment of the text, textual data are not labelled, so the focus is on finding the pattern and gaining insight from that data. Based on the reviewed papers, most researchers had used machine learning techniques to analyse people’s opinions in the business domain. They extract people’s opinions from reviews left on e-commerce platforms. Businesses or products such as skin care, mobile phones, movie reviews, banking and train services have applied machine learning techniques for mining people’s opinions regarding their products and goods. Other than that, machine learning techniques are also used in the health and education domains. For the health domain, the machine learning method has been used to mine people’s opinions on health-related issues such as COVID-19 and medicine reviews. In the education sector, researchers have been more focused on the e-learning environment to analyse student reviews regarding e-learning. Government-related domains, such as politics and the economy, also apply machine learning techniques.

Under supervised learning, machine learning methods include the Naïve Bayes Classifier, Support Vector Machine, Decision Tree and Maximum Entropy. Based on the review articles, most methods employed by the researcher have been Naïve Bayes Classifier and Support Vector Machine. In the transportation domain, Mogaji and Erkan [23] identified the textual data on Twitter that will fall into which sentiments category (positive, negative, or neutral) according to consumer experiences of United Kingdom (UK) train transportation services by using the Naïve Bayes algorithm. Thus, the limitation highlighted by that research was that the automated process was prone to error. It needs the involvement of humans to watch out for that process and stated that human emotion does not fit into just three categories of positive, negative, or natural sentiment. It was different on Naïve Bayes Classifier implemented by Kaur and Kumar [24] to analyse public opinions on a crisis based on the social media platform. That research had enhanced the method by adding other features that is unigram, it helps in detecting sentiment that can provide useful information to the government in managing crisis situations, but researcher had to state on doing the approach comparison research by comparing this method with other approaches such as Support Vector Machine (SVM) in finding the appropriate sentiment classifier performance on natural disaster domain.

In 2017, Sabuj et al. [25] used SVM to mine opinions based on data from the web that resulted in satisfactory results when SVM was applied as a polarity classifier. Based on the accuracy comparison value, they found out that the SVM outperformed the Naïve Bayes. The SVM also was employed by Zhang et al. [26] to explore the negative sentiment tweets on Twitter. Even though that research contributes to identifying the negative features of the text on Twitter, it was observed that a more detailed classification of emotions such as positive was able to be identified by this sentiment analysis method. Ameur et al. [27] used the SVM classifier to determine the polarity of the "positive or negative" classification for comments on Facebook.

Researchers also use or combine more than one machine learning technique. Based on the reviewed article, the Naïve Bayes algorithm and Support Vector Machine method was most used together to extract opinions and sentiments from textual data from various datasets and social media. More than one method became the most used method in machine learning since the outcome of predicted data is accurate. According to research by Dhahi and Waleed [28] that employs Naïve Bayes and SVM as machine learning classifiers to extract sentiment from tweet datasets, they found that Naïve Bayes shows acceptable results. Still, it shows a different result from the research performed in [29], where SVM performed slightly better than NB by adding other features called as stemmed unigram that made the precision value of the SVM method higher than NB. Even though these are the two methods frequently used in mining opinions, other methods such as the maximum entropy and decision tree also have been employed to determine the positive and negative opinions based on a textual dataset but because of the lack of result accuracy. In 2019, Elhadad et al. [30] proposed an efficient approach in handling Tweets, in Arabic and English languages, with different processing techniques, such as Decision trees and Naïve Bayes. It was identified that the Decision Tree gets the least value on accuracy, and precision acts as a performance measure on those methods.

The supervised learning technique had limitations because machine learning applies the method of training and testing. As a result, researchers need to conduct the time-consuming training phase to get the result. Moreover, a training dataset and testing dataset are usually prepared by employing existing datasets due to requirements in the machine learning method that needs labelled data to train classifiers. It is necessary for datasets used in the experiment to be labelled with an opinion flag. For example, Twitter and movie review datasets are embedded with positive and negative reviews that resulted in the datasets made available with polarity labels (positive, negative, and neutral). Since the classification of sentiments within sentences usually uses machine learning algorithms, thus the input dataset is desired to be labelled.

Random forest, a semi-supervised learning technique, is another method that researchers have implemented in previous studies. In 2018, Khanvilkar and Vora [31] proposed the use of the random forest as the classification for sentiments on product reviews. The researchers have stated that the random forest machine learning algorithm will help improve sentiment analysis for product recommendations using multiclass classification. In 2020, Suganya and Vijayarani [32] used the deep learning method in opinion mining. They found that the time taken of execution of random forest was more than the CNN, one of the deep learning methods. Deep learning is a subfield of machine learning that employs deep neural networks. Recently, deep learning algorithms have been widely used in opinion mining. This section provides an overview of papers that have applied deep learning for opinion mining. Deep learning is one of the methods of semi-supervised learning. Imran et al. [33] used the deep learning method in the health domain. The deep long short-term memory (LSTM) was employed to detect the polarity and emotion on COVID-19 related tweets. That article successfully observed and detected the correlation between sentiments and emotions of people from within neighbouring countries amidst coronavirus (COVID-19) outbreak from their tweets but had some limitations on understanding the tweet context.

Other researchers have also used deep learning methods (such as CNN and LSTM) for analysing the emotional reactions to events of mass violence as well as to enhance the capability and accuracy of the opinion mining method based on a textual dataset by considered properties of users and events, generalized conclusions using several events [34]. The researcher observed that the CNN model was an appropriate method with meaningful and representative features for prediction. The deep learning method proved to be capable of classifying opinions into positive, negative, and other emotions. However, these supervised algorithms requiring a large dataset to predict the accurate result make this method time-consuming [35].

**Fig. 6:**
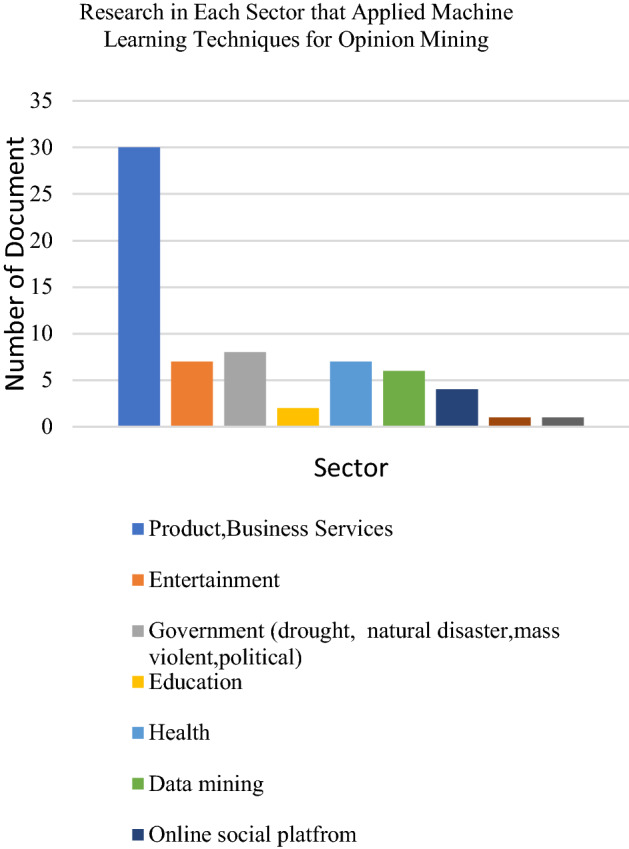
Chart on the application of machine learning techniques for Opinion mining

**Fig. 7:**
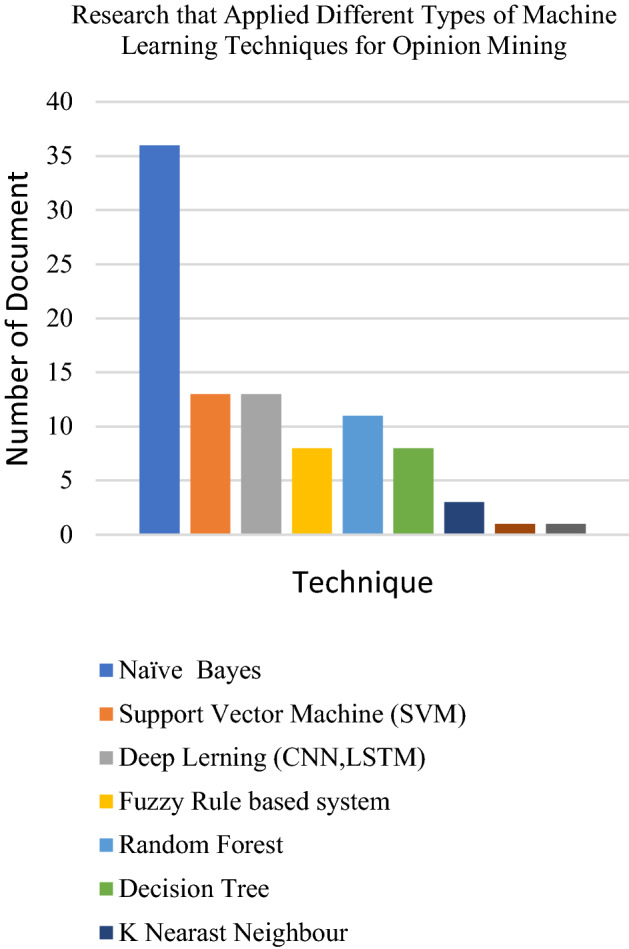
Chart of machine learning techniques for Opinion mining

**Fig. 8:**
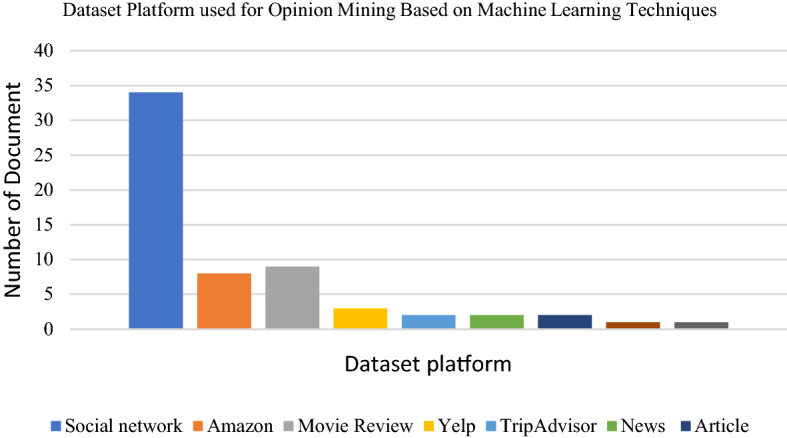
Dataset platforms used for opinion mining based on machine learning techniques

Datasets from social media platforms such as Twitter, Facebook and Tumblr are the textual datasets used by researchers. The text mostly consists of user comments, reviews or related research topic words on businesses, products, or events. Researchers have also used existing datasets in cyberspace websites such as IMDB and Amazon review datasets. Several researchers have also applied other dataset platforms such as text in the news, articles and emails. The following Figs. 6, 7 and 8 presents the distribution of articles according to application, technique and dataset platforms. The machine learning techniques used in opinion mining from the text are summarized in the Tables 1, 2, 3, 4, 5, 6 below.

Table 1 summarizes the Naïve Bayes/Bayesian techniques used in opinion mining based on text.

**Table 1:** Summary of Naïve Bayes/Bayesian techniques used in opinion mining from text

ML methods	Reference	Objectives	Materials	Output
NB	[6]	To present a continuous Naïve Bayes learning framework for e-commerce product review sentiment classification	E-commerce review and Cornell Movie review dataset	Positive, negative and neutral
	[24]	To develop a workflow for applying sentiment analysis in detecting public emotions in natural disaster crises	Twitter (Kashmir Floods)	Negative, positive and neutral
	[23]	To explore consumer attitudes and experiences of "train operating companies."	Twitter (tweets on train operating companies)	Positive or negative
	[36]	To access and classify Tweets for counter violent extremism and the spread of extremist content on Twitter	Twitter Data	Positive, negative and neutral
	[37]	To investigate tourist emotions on their travel experiences targeting Gatlinburg, Tennessee	Online reviews of Tripadvisor	Emotions (anger, disgust, fear, joy, sadness and surprise)
	[21]	To analyse every food review of the user and classify if it is positive, negative or neutral	McDonald’s dataset is customer reviews	Positive, negative and neutral
	[38]	To monitor public opinion on trending topics on the social media platform	Twitter	Positive, negative or neutral
	[39]	To perform aspect-based sentiment analysis by filtering statements from the review pertinent and extracting sentiments from the reviews, and associating them with corresponding aspect categories	Amazon movie review dataset	Positivity or negativity
NB + SVM	[40]	Analyse opinions on smartphone reviews	Smartphone reviews	Positive and negative
	[41]	Survey different types of sentiment analysis methods based on cryptocurrencies topic	Twitter	Positive, neutral and negative
	[42]	Identify the levels of positive and negative emotion in messages	Twitter comment,	unrelated, neutral, negative and positive messages
	[29]	To develop a polarity detection system on textual movie reviews in Bangla	Text movie review in Bangla	Positive or negative
	[28]	To implement a combination of user behaviour, semantic and lexical features together for finding polarity emotions of Tweets	Twitter	Positive and negative
	[43]	To analyse and consider traffic jam events where traffic will be able to move or will not be able to move	Twitter (traffic jams)	Positive, negative or neutral
NB + SVM + DBN	[44]	To classify a Malay sentiment by proposing a classification model to improve classification performances	Online blogs and forums of Malaysian website	Positive and negative
NB + DT	[45]	Find the polarity of any sentence by analysing the opinion of that particular sentence	Hindi sentences and reviews	Positive, neutral and negative
NB + DT	[30]	To apply an efficient processing approach in handling Tweets, in both Arabic and English languages	Tweets Dataset (ASTD) and Restaurant Reviews Dataset (RES) Stanford Twitter dataset, Twitter US Airline Sentiment dataset and the Uber Ride Reviews dataset	Positive, negative and neutral
NB + ME	[46]	To evaluate the accuracy of combining different parameters of machine-learning algorithms for consumer products	Twitter	Positive or negative
NB + ME + SGD + SVM	[47]	To classify human sentiment-based movie reviews using various supervised machine learning algorithms To examine the accuracy of different methods	Internet Movie Database (IMDB)	Positive, negative and neutral
NB + LR + DT	[48]	To perform tweets classification with the help of Apache Spark framework	Twitter dataset (Kaggle and Twitter Sentiment Corpus)	Positive, negative or neutral
CNN + NB + J48 (DT) + BFTree, OneR + LDA + SVM	[49]	Introduce and examine the proposed technique with Convolution Neural Network used for text classification	IMDB movie portal, Amazon product reviews	Positive negative and neutral
SVM + NB + RF	[20]	To provide sentiment mining in extracted sentiment from Twitter Social App for analysis of the current trending topic in India and its impact on different sectors of the Indian economy	Tweets	Positive, negative and neutral
SVM + NB + RF	[50]	Mining consumer reviews with a machine learning approach by converting reviews into vector representations for classification	Amazon review dataset	Positive or negative
Multinomial NB + SVM	[51]	Develop an efficient review classification	Reviews TripAdvisor dataset	Positive and negative
SVM + Multinomial NB + LR + RF	[52]	To develop a clinical decision support system for the personalised therapy process	Drug review dataset	Positive, negative or neutral
SVM + CRF + Multinomial NB	[53]	To present an ensemble framework of text classification which reviews products	Twitter and product review	Positive and negative
Multinomial NB + SVM + LR	[54]	To compare the performance of different machine learning algorithms in performing sentiment analysis of Twitter data	Twitter	Positive or negative
DT + Multinomial NB + SVM	[55]	To investigate three approaches for emotion classification of opinions in the Thai language	Customer reviews of cosmetics Thai	Positive and negative
SVM + Multinomial NB + DNN	[56]	To compare multiple state-of-the-art models capable of classifying game reviews as positive, negative or neutral	Games reviews	Positive, neutral and negative
Bernoulli NB + SVM + RF + NNs + LR	[57]	To present a comparison among several sentiment analysis classifiers in the learning environment	Twitter (educational opinions in an Intelligent Learning Environment)	Emotions positive or negative, engagement, excited, boredom and frustration
LR + k-NN + SVM + DT + RF + Ada Boost + Gaussian NB	[58]	To analyse the reviews posted by people at four different product websites	Amazon reviews, Yelp reviews, IMDB reviews, Indian Airlines reviews	Positive and negative

Table 2 summarizes the Support Vector Machine (SVM) techniques used in opinion mining based on text.

**Table 2:** Summary of Support Vector Machine (SVM) techniques used in opinion mining from text

ML method	Reference	Objectives	Materials	Output
SVM	[25]	Design opinion classifier for classifying opinions from Bangla text data	Twitter text, English, Bangla	Positive and negative
SVM	[59]	To extract multi-class emotions from Malayalam text using the proposed approach	Malayalam text	Emotions (joy, sadness, anger, fear, surprise or normal)
SVM	[60]	To determine the expressed sentiment towards a specified aspect category in a given sentence	Yelp restaurant reviews corpus	Negative, positive and neutral
SVM	[61]	To propose and analyse new emotion identification method based on online medical knowledge-sharing community	Medical service comments	Positive and negative
SVM	[26]	To address the challenge of analysing the features of negative sentiment tweets	Twitter (TREC Microblog Track 2013)	Negative
SVM	[62]	To rank colleges based on a single feature, multiple features and no feature	Twitter (colleges)	Positive, negative or neutral sentiment
SVM	[27]	To determine the polarity of Facebook comments “positive or negative”	Facebook dataset (Tunisian political pages)	Positive and negative
SVM + RF	[31]	Determines polarity of reviews given by users and provide recommendation list	Twitter stream	Positive and negative
SVM + ANN + RF	[7]	To evaluate the thoughts of users in the IMDB movie reviews on tweets obtained from different outlets	IMDB dataset, Review Movie	Positive and negative
SVM + CRF + Multinomial NB	[53]	To present an ensemble framework of text classification which reviews products	Twitter and product review	Positive and negative
SVM + NB + RF	[50]	Mining consumer reviews with a machine learning approach by converting reviews into vector representations for classification	Amazon review dataset	Positive or negative
SVM + Multinomial NB + DNN	[56]	To compare multiple state-of-the-art models capable of classifying game reviews as positive, negative or neutral	Games reviews	Positive, neutral and negative
NB + ME + SGD + SVM	[47]	To classify human sentiment-based movie reviews using various supervised machine learning algorithms To examine the accuracy of different methods	Internet Movie Database (IMDB)	Positive, negative and neutral
KNN + SVM + RF	[63]	To classify sentiments into positive, negative or neutral polarity using a new similarity measure	Stanford Twitter dataset	Positive, negative or neutral polarity
SVM + Multinomial NB + LR + RF	[52]	To develop a clinical decision support system for the personalised therapy process	Drug review dataset	Positive, negative or neutral
NB + SVM + DBN	[44]	To classify a Malay sentiment by proposing a classification model to improve classification performances	Online blogs and forums of Malaysian website	Positive and negative
Fuzzy rule + SVM + ME	[64]	Social Media data for decision making to purchase and recommend products online	Twitter text reviews	Positive and negative

Table 3 summarizes the Random Forest (RF) techniques used in opinion mining based on text.

**Table 3:** Summary of random forest (RF) techniques used in opinion mining from text

ML method	Reference	Objectives	Materials	Output
RF	[65]	Conducting sentiment analysis of captions on public libraries on Instagram To understand readers and help libraries deliver better services	hashtags #reading and #read public content on Instagram	Positive and negative
RF	[66]	To perform sentiment analysis of real-time 2019 election twitter data	Twitter data (Indian Elections)	Positive and negative
SVM + Multinomial NB + LR + RF	[52]	To develop a clinical decision support system for the personalised therapy process	Drug review dataset	Positive, negative or neutral
Bernoulli NB + SVM Linear SCV + RF + NNs + LR	[57]	To present a comparison among several sentiment analysis classifiers in the learning environment	Twitter (educational opinions in an Intelligent Learning Environment)	Emotions positive or negative, engagement, excited, boredom and frustration
ANN + RF + SVM	[67]	To presents emotion recognition in email texts	Email text	Neutral, happy, sad, angry, positively surprised and negatively surprised
SVM + ANN + RF	[7]	To evaluate the thoughts of users in the IMDB movie reviews on tweets obtained from different outlets	IMDB dataset, Review Movie	Positive and negative
KNN + SVM + RF + CNN	[32]	To extract content from an e-commerce website and analyse it using opinion or sentiment analysis classification model	product review comments (online shopping websites) (Amazon, Flipcart and Snapdeal)	Positive, negative or neutral
LR + k-NN + SVM + DT + RF + Ada Boost + Gaussian NB	[58]	To analyse the reviews posted by people at four different product websites	Amazon reviews, Yelp reviews, IMDB reviews, Indian Airlines reviews	Positive and negative
SVM + NB + RF	[20]	To provide sentiment mining in extracted sentiment from Twitter Social App for analysis of the current trending topic in India and its impact on different sectors of the Indian economy	Tweets	Positive, negative and neutral
SVM + NB + LR + RF	[50]	Mining consumer reviews with a machine learning approach by converting reviews into vector representations for classification	Amazon review dataset	Positive or negative

Table 4 summarizes the Decision Tree (DT) techniques used in opinion mining based on text.

**Table 4:** Summary of decision tree (DT) techniques used in opinion mining from text

ML method	Reference	Objectives	Materials	Output
NB + DT	[45]	Find the polarity of any sentence by analysing the opinion of that particular sentence	Hindi sentences and reviews	Positive, neutral and negative
k-NN + Gaussian NB + Multinomial NB + Bernoulli NB + SVM + RBF + DT	[68]	Provide a method to overcome the problem of lower accuracy in cross-domain sentiment classification	Amazon (hotel reviews obtained from TripAdvisor reviews)	Positive or negative
CNN + NB + BFTree, OneR + LDA + SVM	[49]	Introduce and examine the proposed technique with Convolution Neural Network used for text classification	IMDB movie portal, Amazon product reviews	Positive negative and neutral
NB + LR + DT	[48]	To perform tweets classification with the help of Apache Spark framework	Twitter dataset (Kaggle and Twitter Sentiment Corpus)	Positive, negative or neutral
LR + k-NN + SVM + DT + RF + Ada Boost + Gaussian NB	[58]	To analyse the reviews posted by people at four different product websites	Amazon reviews, Yelp reviews, IMDB reviews, Indian Airlines reviews	Positive and negative
NB + DT	[30]	To apply an efficient processing approach in handling Tweets, in both Arabic and English languages	Tweets Dataset (ASTD) and Restaurant Reviews Dataset (RES) Stanford Twitter dataset, Twitter US Airline Sentiment dataset and the Uber Ride Reviews dataset	Positive, negative and neutral
DT + Multinomial NB + SVM	[55]	To investigate three approaches for emotion classification of opinions in the Thai language	Customer reviews of cosmetics Thai	Positive and negative

Table 5 summarizes the Deep learning techniques used in opinion mining based on text.

**Table 5:** Summary of Deep learning techniques used in opinion mining from text

ML method	Reference	Objectives	Materials	Output
LSTM + DNN	[33]	Analyse the reaction of citizens from different cultures regarding novel Coronavirus Define people’s sentiments about subsequent actions taken by different countries	Sentiment140 and Emotional Tweets datasets	Positive or negative, Emotions (joy, surprise, sadness, fear, anger and disgust)
CNN + biLSTM BERT	[34]	Investigate the emotional reactions on Twitter to mass violent events and derive conclusions from it	Twitter mass shootings	Emotions (anger, fear, sadness, disgust and surprise)
LSTM (biLSTM) + GRU	[69]	Classify longer sentences with polarity from a huge amount of data	Articles, forums, consumer reviews, surveys, blogs, Twitter and WhatsApp chat	Emotions (sadness, joy, surprise, anger)
CNN + NB + J48 + BFTree, OneR + LDA + SVM	[49]	Introduce and examine the proposed technique with Convolution Neural Network used for text classification	IMDB movie portal, Amazon product reviews	Positive negative and neutral
CRF	[70]	Extract opinion holder, opinion target, opinion polarity from news articles	News articles	Positive and Negative
LDA	[71]	To study the public perception of social distancing through large-scale discussions on Twitter	Tweets on social distancing hashtags	Positive, negative or neutral
NNs	[72]	Evaluate the current potential of sentiment analysis and machine learning To extract the importance of the reported results and conclusions of randomised trials on stroke	Text abstracts of 200 articles	Negative result
RNN	[73]	To identify the sentiment polarity and predominant emotions in tweets about the COVID-19 pandemic	Tweets matching hashtags (COVID-19-related tweets)	Positive, negative or neutral and emotions (anger, disgust, fear, joy, sadness or surprise)
ML-KNN	[74]	To design a multi-label learning approach in detection of multiple emotions in online social network	Twitter	Emotions (joy, sadness, surprise, anger, fear and disgust)
ANN + RF + SVM	[67]	To presents emotion recognition in email texts	Email text	Neutral, happy, sad, angry, positively surprised and negatively surprised
NB + SVM + DBN	[44]	To classify a Malay sentiment by proposing a classification model to improve classification performances	Online blogs and forums of Malaysian website	Positive and negative
CNN	[75]	To provide a CNN-based sentiment classification approach that can be used in Android applications to classify reviews from various streaming services like Netflix and Amazon without server-side APIs	Review data mobile environment (IMDB and Rotten Tomatoes data sets)	Positive and negative
SVM + ANN + RF	[7]	To evaluate the thoughts of users in the IMDB movie reviews on tweets obtained from different outlets	IMDB dataset, Review Movie	Positive and negative
KNN + SVM + RF + CNN	[32]	To extract content from an e-commerce website and analyse it using opinion or sentiment analysis classification model	product review comments (online shopping websites) (Amazon, Flipcart and Snapdeal)	Positive, negative or neutral
SVM + CRF + Multinomial NB	[53]	To present an ensemble framework of text classification which reviews products	Twitter and product review	Positive and negative
NNs	[76]	To apply neural network-based methods for opinion mining from the social web in the health care domain	Drug review dataset	Positive, negative or neutral
SR-LSTM + NB + SVM	[77]	To introduce a neural network model with two hidden layers to learn continuous document representation for sentiment classification	IMDB is a large movie review dataset, Yelp 2014 and Yelp 2015 are two restaurant review datasets	Positive and negative
BERT LSTM	[78]	To present the results from applying BERT, a transfer learning method, in Vietnamese text classification	VLSP 2018, Hotel and Restaurant Vietnamese	Positive, negative or neutral
BPNN + SVM + LDA	[79]	To analyse the twitter dataset of particular policies and finding its polarity of sentiment	Twitter text	Positive and negative

Table 6 summarizes the Deep learning techniques used in opinion mining based on text.

**Table 6:** Summary of logistic regression used in opinion mining from text

ML method	Reference	Objectives	Materials	Output
SVM + Multinomial NB + LR + RF	[52]	To develop a clinical decision support system for the personalised therapy process	Drug review dataset	Positive, negative or neutral
Bernoulli NB + SVM Linear SCV + RF + NNs + LR	[57]	To present a comparison among several sentiment analysis classifiers in the learning environment	Twitter (educational opinions in an Intelligent Learning Environment)	Emotions positive or negative, engagement, excited, boredom and frustration
NB + LR + DT	[48]	To perform tweets classification with the help of Apache Spark framework	Twitter dataset (Kaggle and Twitter Sentiment Corpus)	Positive, negative or neutral
LR + k-NN + SVM + DT + RF + Ada Boost + Gaussian NB	[58]	To analyse the reviews posted by people at four different product websites	Amazon reviews, Yelp reviews, IMDB reviews, Indian Airlines reviews	Positive and negative
Multinomial NB + SVM + LR	[54]	To compare the performance of different machine learning algorithms in performing sentiment analysis of Twitter data	Twitter	Positive or negative
SVM + NB + LR + RF	[50]	Mining consumer reviews with a machine learning approach by converting reviews into vector representations for classification	Amazon review dataset	Positive or negative

### Lexicon-based approach

Another method for opinion mining or sentiment analysis would be the lexicon-based approach. The lexicon-based approach employs a dictionary that incorporates the polarity of the word inside it. If a word is found in a text, it is compared to a word in the dictionary, and the sentiment score is applied. The lexicon-based approach is used to determine sentiment, which is then computed by the overall polarity included in a text.

The lexicon-based approach can be classified under the unsupervised method. This method involves counting the positive and negative words related to the data. This method must also implement a lexicon, known as dictionaries. The dictionaries can be created manually or automatically from existing dictionaries. The difference between this method from machine learning is that it does not depend on or require any training data since it only employs the dictionary.

Through this research, 23 articles that use the lexicon-based approach for opinion mining or sentiment analysis were reviewed and implemented this approach to conduct emotion analysis to determine the sentiments and opinions of the textual dataset. Based on the reviewed articles, most research utilises the lexicon-based approach to extract opinions on business, products and e-commerce domains. Half of the reviewed articles had used a lexicon-based approach for analysing sentiments and emotion data on products and services such as cameras, mobile phones, laptops, tablets, TVs, video surveillance devices and movie reviews. Several types of research have also focused on education and health domains. Researchers employ this approach to analyse people’s opinions on a certain topic related to government issues such as political issues, election-related matters as well as environmental and energy resources.

For the lexicon-based approach, two techniques have been used by researchers: the dictionary-based approach and the corpus-based approach. The first technique, the dictionary-based approach, is employed to pinpoint the opinion words and their polarities.

Usually, to determine sentiments or opinions of the word, the dictionary-based approach is used where synonyms, antonyms and hierarchies in existing lexicons with sentiment information are found. In the existing lexicon, there are three numerical sentiment scores used: Obj(s), Pos(s) and Neg(s), which signify the Objective, Positive and Negative synset. This method is utilised to tag the polarity value with the sentiment dictionary, also known as the sentiment lexicon. Fernández-Gavilanes et al. [35] had employed the dictionary-based approach to detect opinions on online text such as tweets and reviews. The researcher stated the advantages of this method that can be applied to subject domains other than the domain it was designed for and fix some generic lexicon issues on not context-based by employing a context-based algorithm that helps create a dictionary/lexicon based on a particular context.

Abd et al. [80] further aimed to recognise the emotional segmentation of a movie reviewer based on the entertainment domain by using this approach to extract sentiments from a given text and classify them. Lexicon based approach helps them achieve a significant result by identifying the contextual polarity for a large subset of sentiment. It was suggested to apply this dictionary idea with machine learning to enhance the accuracy of the result. Also, the researcher had implemented existing dictionaries such as Wordnet and SentiWordNet.

The most used lexicon for the lexicon-based approach, according to the papers reviewed is SentiWordNet. SentiWordNet is the dictionary mostly employed for opinion mining. SentiWordNet is a lexical resource derived from WordNet which assigns numerical values to each synset, representing the scores of positivity, negativity or objectivities [81]. Each score has a value between 0 and 1, and the sum of positivity, negativity, or objectivity scores is 1. For example, Khan et al. [82] used the SentiWordNet to create their sentiment dictionary capable of enhancing the polarity classification in sentiment analysis based on movie review dataset and increasing the capability of SentiWordNet.

Even though SentiWordNet is the most frequently used because of the improvement of its usability in opinion mining. Other lexicons, such as MPQA, Wordnet, Vader, and Pattern lexicon was less selected by researchers because of their lack of capabilities in opinion classification. However, it is still able to be applied by researchers for opinion mining. For instance, Wordnet was used as an association list for the opinion classifier of user comments in online media platforms. It was observed that the dictionary enables the classification of irrelevant comments with a high score of precision value but less accuracy in finding relevant and positive comments [83]. Recently, Dey et al. [84] used the Vader lexicon, another type of dictionary, compared with other classification methods such as n-gram based SO-CAL approach and Senti-N-Gram lexicon based on those methods in determining the polarity of opinions in a movie review. The results show, the Vader lexicon got less score on accuracy between those two methods.

Other researchers also used an existing dictionary, called the NRC emotion lexicon, for classifying the opinion or polarity according to emotions. The NRC emotion lexicon is a list of words and their corresponding emotions. Eight emotions (fear, sadness, disgust, anger, trust, surprise, anticipation, and joy) and two sentiments (positive and negative) are included in this NRC emotion lexicon. In 2019, Swain and Seeja [85] employed this lexicon to develop a web-based application that may predict polarity and emotion based on data from Twitter. That lexicon helps classify people’s opinions such as emotions (joy, sadness, disgust, anticipation, trust, fear, surprise, anger, positive and negative) and helps government analyse peoples’ perception with sentiment analysis. However, the web application was only an experiment on the related Tweet on demonetization in India, not in other domains or issues.

As previously mentioned, the other method in the lexicon-based approach is the corpus-based approach. It works when a new sentiment word is recognised based on its mutual relationship. It exploits co-occurrence patterns of words found in unstructured textual documents. In the corpus-based approach, new sentiment words are recognised based on their relationship with other words. This approach can use an existing dictionary or generate a new lexicon based on the research domain to clarify the opinion or sentiment. Deng et al. [86] had developed a corpus according to the vital research topic regarding social media to be used to extract people’s opinions. The observation of result use for this approach is helpful in domain-specific sentiment classification that is implemented in existing sentiment lexicons. Still, the effectiveness of that method was dependent on the heuristic limitation, which is the frequently co-occurring words are likely to have similar sentiment orientation. The corpus-based approach can be used to analyse the diversity of online opinions that have a potential impact in commercial, industrial and academic environments. However, the extraction and processing of opinions are complex and difficult tasks.

The lexicon-based approach is dependent on lexical resources, and the overall success of the technique is highly dependent on the quality of the lexical resources. It is based on the polarity of a line of text, which may be determined by the polarity of the words that constitute that text. This approach is not meant to address all aspects of language, particularly slang, irony, and negation, because of the complex nature of natural language. Using sentimental language is insufficient. Some issues do exist, such as the fact that some words have varying meanings depending on the application, that some phrases including emotion words might not express any opinion or emotion. From there, this technique has a low recall and a low accuracy. However, the lexicon-based approach has its own advantages, including the following: it can simply count positive and negative words, it is adaptable to many languages and speeds up analysis, and it is fast in terms of processing because it does not require training for its data. The following table displays a summary of review papers on the lexicon-based approach used in opinion mining.

**Fig. 9:**
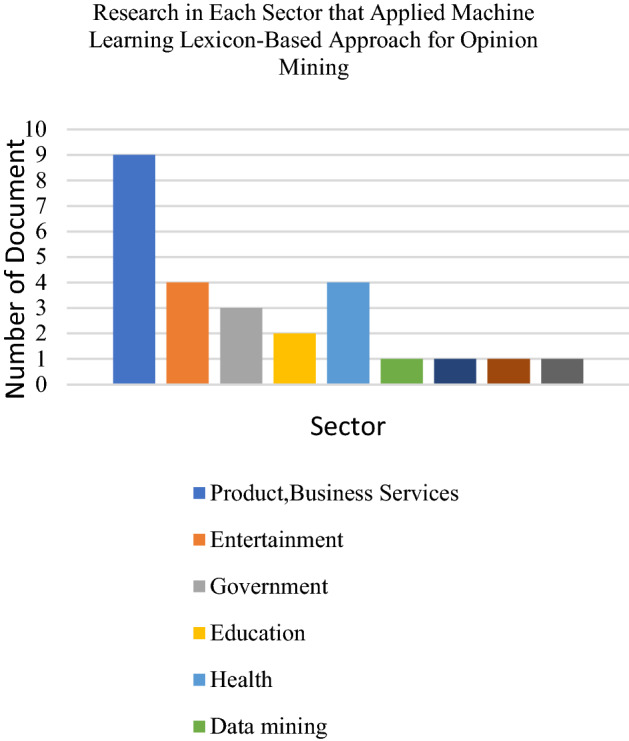
Chart on application of lexicon-based approach for opinion mining

**Fig. 10:**
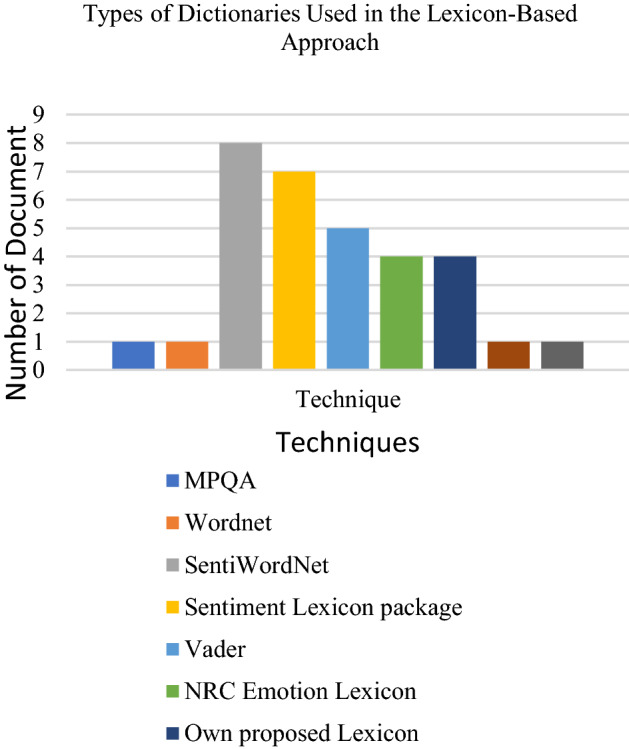
Chart on dictionaries used in lexicon-based approach for opinion mining

**Fig. 11:**
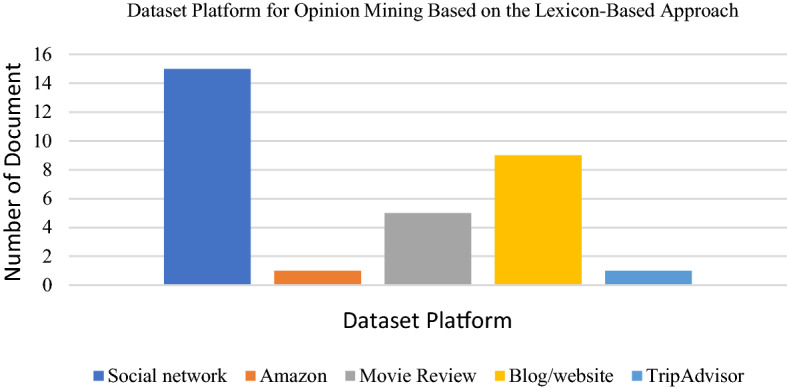
Chart of dataset platforms used in lexicon-based approach for opinion mining

We found that the most applied dataset platform for the lexicon-based approach is the Twitter dataset. Next would be the movie review dataset. Researchers also frequently use other datasets from websites such as online shopping sites. Facebook platforms and blogs have been somewhat utilised depending on the specific research domain. The following Figs. 9, 10 and 11 presents the distribution of articles according to their application, technique and dataset platforms. Tables 7 and 8 below show the detail of articles that employ the Dictionary based approach and Corpus-based approach.

**Table 7:** Summary of the lexicon-based approach (dictionary based approach) used for opinion mining

Reference	Objectives	Lexicon type	Materials	Output
[35]	To predict whether an online text expresses positive, negative or neutral sentiments without the need for supervision	Dictionary-based approach	The Cornell Movie Review dataset, The Obama-McCain Debate dataset, the SemEval-2015 dataset	Positive, negative or neutral
[82]	To improve the SWN performance by building a new lexical resource named SentiMI	SentiMI based classification, SentiWordNet	Movie review dataset	Positive, negative and objective
[85]	To present a web-based system known "TweeSent" that can estimate the polarity and emotion of tweets based on their input data from Twitter	NRC emotion lexicon	Tweets from Twitter	Joy, happiness, sadness, anger, trust, surprise, anticipation, fear, positive and negative
[87]	To classify movie reviews into positives, negatives and neutral polarity	The lexicon that has been published by Hu and Liu (2004)	Twitter data	Positives, negatives and neutral
[88]	To improve SentiWordNet performance and propose a complete sentiment analysis and classification framework according to SentiWordNet based vocabulary	SentiWordNet based classification	Large movie review dataset, Cornell movie review dataset, multi-domain sentiment datasets	Positive, negative or neutral
[89]	To investigate Alaskans’ perceptions and opinions on various energy sources and, in particular, clean energy sources	Subjectivity lexicon of English adjectives called ADJLex	Twitter data (Alaskans’ review) on energy consumption	Positive, neutral and negative
[80]	To recognise the emotional segmentation of a movie reviewer by extracting the sentiments from a given text and classifying them	Dictionary-based methods	Text movie review (IMDB)	Positive and negative
[90]	To automatically analyse student feedbacks (known as OMFeedback)	Vader Sentiment Intensity Analyser database of English sentiment words (Vader Lexicon)	Feedback	Positive, negative and neutral
[91]	To extract and classify sentiments and emotions from 141,208 headlines of global English news sources regarding the coronavirus disease (COVID-19)	NRC emotion lexicon, R package “sentiment”	English Headlines news sources	Positive, negative and neutral
[92]	To identify the public opinion of Filipino Twitter users concerning COVID-19 in three different timelines	Lexicon-based Approach R package “sentiment dictionary”	Twitter textual (COVID-19)	Positive, negative, joy, sadness, fear, anticipation, anger, trust, surprise, disgust
[93]	To classify user reviews and use co-occurrence analysis to identify passengers’ concerns on different aspects of service in the aviation industry	Vader and Pattern lexicons	Reviews on SKYTRAX	Positive, negative and neutral
[94]	To study people’s reactions and emotions regarding Trump’s primary debates	R package “sentiment dictionary”	Tweets regarding the Trump Republican primary debate	Negative or positive
[95]	To illustrate and analyse the emotional sentiment of the campaign speeches of the two main candidates of 2016 US presidential elections	Word-Emotion Association Lexicon	Text files of American Presidency Project website	Negative and Positive
[96]	To estimate the reputation polarity of tweets	RepLab 2013 collection	Twitter data in English and Spanish	Positive, negative or neutral
[83]	To categorise YouTube comments based on content relevance	Wordnet	Keenformatics	Relevant, irrelevant, positive and negative
[97]	To correlate the distinct twitter comments of statesmen of distinct countries for having concrete knowledge on the application of drugs to patients attacked by COVID-19	TextBlob lexicon	Twitter	Positive and negative

**Table 8:** Summary of the lexicon-based approach (Corpus based approach) used for opinion mining

Reference	Objectives	Lexicon type	Materials	Output
[98]	To introduce SmartSA, a lexicon-based sentiment classification system for social media genres	Hybridise a general-purpose lexicon, SmartSA, SWN	Twitter, Digg, MySpace	Positive and negative
[99]	To improve the detection of emotional state of patients in Brazilian online cancer communities by using the proposed approach	SentiHealth-Cancer (SHC-pt)	Facebook	Positive, negative or neutral
[100]	To present the results of the systematic analysis of opinion mining (OM) for YouTube comments	Italian sentiment dictionary from the SentiWordNet sentiment lexicons and the MPQA Lexicon	Review from videos of products, English and Italian	Positive, negative or neutral
[86]	To learn sentiment words based on both content domain and language domain	Corpus-based lexicon generation method	Twitter stock market	Positive and negative
[101]	To extract aspects, classify aspect-related sentiment and generate an aspect-level summary	Hybrid sentiment classification scheme, lexicon-based (corpus-based approach) SentiWordNet lexicon	Product reviews	Positive and negative
[102]	To detect sentiment out of textual snippets which express people’s opinions in different languages by proposed methodology	Hybrid approach lexicon Greek Sentiment Lexicon, NRC Word-Emotion Association Lexicon (EmoLex)	Online user reviews in both Greek and English (Greek e-shopping site with various products)	Positive or negative
[97]	To correlate the distinct twitter comments of statesmen of distinct countries for having concrete knowledge on the application of drugs to patients attacked by COVID-19	TextBlob lexicon	Twitter	Positive and negative

### Hybrid approach

Researchers have implemented the hybrid approach in performing opinion mining. The hybrid approach has been implemented to cover up the incapability’s of machine learning and lexicon-based approach by combining two or more methods to achieve better accuracy in extracting and classifying people’s opinions. Based on the reviewed research papers, most researchers use the hybrid approach for opinion mining of products and businesses such as cameras, hairdryers, aircraft, IKEA products and the stock market. It has been further employed in the education and health sectors. Also, we found that the most used machine learning techniques in the hybrid approach are the Naïve Bayes Classifier and Support Vector Machine. Other methods such as the Fuzzy rule-based system, random forest, and deep learning have also been combined with the lexicon-based approach. The most used lexicon/dictionary in the hybrid approach is SentiWordnet, where 16 papers had implemented this lexicon. Other lexicons such as Wordnet, Pattern lexicon, VADER, and NRC Emotion lexicon were also used in this hybrid approach. Mahajan and Rana [103] had applied eight emotions from the NRC emotion lexicon to quantify public emotion. Several types of research have also used existing sentiment lexicon packages (such as “sentiment r”) and existing dictionaries (such as English sentiment dictionary and Dutch sentiment dictionary). Also, many articles used their own lexicon and combined it with the machine learning method.

Based on research in the business/tourism domain by Chen et al. [104], the hybrid approach was implemented to construct a tourism sentiment model to achieve text sentiment classification that accurately understood tourist emotions and benefits management and business operations domain. The first method was using the dictionary-based method, which is one of the lexicon-based approaches, to calculate the sentiment value of a single-sentence text. For the second method, the Naïve Bayes machine learning algorithm was used to construct the classifier. Researchers observe that only using a dictionary method has an unacceptable effect on corpus classification. When the NB classifier is used to classify the corpus, the effect will be fixed and improved. Keyvanpour et al. [105] had implemented the hybrid approach based on lexicon and machine learning to recognize people’s opinions on social networks. The polarity of opinions toward a target word was determined using a method based on the lexicon approach. The textual features of words, sentences, and opinions were analysed and classified using the deep learning method (Neural-fuzzy network). The result from that method had been compared with other supervised methods and found that this method’s speed is slightly slower than other methods because the meta-heuristic algorithm calculates the cost of each member of the population repeatedly using a cost function until determining optimum values for the parameters.

Different from the research by Hamad et al. [106] used more than one machine learning technique in their hybrid approach for the research that was based on product reviews in the social network. The flow of the approach is identical with the lexicon-based approach is usually the first phase employed lexicon dictionary to determine the sentiment polarity of the sentence, but the machine learning method is used to find and classify the accurate label of polarity and emotion of sentences was different. This research employs the ZeroR, NB, K-NN and Linear SVM as the machine learning method. This approach was compared with some approaches to measure the performance of K-NN, NB and SVM classifiers. It was observed that the K-NN, NB, SVM, and ZeroR have a reasonable accuracy rate. However, the K-NN has outperformed the NB, SVM, and ZeroR based on the achieved accuracy rates and trained model time. The K-NN has achieved the highest accuracy rates of 96.58% and 99.94% for the iPad and iPhone emotion data sets. Despite the result, the researcher highlights the challenge for this approach, such as control of implicit attributes of products, building a summary of opinions based on attributes of products, and dealing with negation opinion expressions. The following Tables 9 and 10 presents a summary of review papers on the hybrid approach used in opinion mining.

**Table 9:** Summary of hybrid approach (combination only one of machine learning method with lexicon-based approach)

Reference	Objective	Method used in hybrid approach for opinion mining	Materials	Output
[107]	To perform sentiment analysis in customer review real word data	K-Mean Clustering + MPQA	Amazon review texts	Subjective expressions, positive, negative, neutral
[108]	To determine sentimental state of a person or a group of people using data mining	NB + lexicon-based analyser R platform	Twitter tweets	Emotions (anger, fear, disgust, surprise, happiness, and sadness), polarity (positive, negative, neutral)
[109]	To address the problem of estimating public opinion in social media content by proposing an aspect-based opinion mining model	NB + Wordnet	Online camera reviews	Positive, negative, neutral
[105]	To determined polarity of opinions toward a target word To analyse and classify opinions	Neural-fuzzy network + SentiStrength data	Twitter	Positive polarity and negative polarity
. [110]	To build a customisable platform that collects the stream of relevant tweets generated by users, store them and do the sentiment analysis	SVM + SWN	Twitter, Heathrow and aircraft noise	Positive, negative or neutral
[111]	To classify tweets into three classes (positive, negative, neutral) using hybrid approach based on particular domain	Fuzzy logic + SentiWordNet	Tweets according or linked to a product, a hashtag or a movie review	Positive, negative or neutral
[112]	To find the scores of opinions from people’s reviews and derive conclusions	SVM + Wordnet	A movie review dataset has been collected from Twitter reviews	Negative and positive
[104]	To construct tourism emotion model	NB + sentiment dictionary constructed by Chen Bing	Microblog travel text online commentary	Positive, negative
[113]	To conduct emotion analysis in e-learning materials	SVM + SentiWordNet	E learners’ comments	Positive, negative, or neutral
[114]	To focus on sentiment analysis in financial newswire text To classify sentiment expressed about certain companies in financial news articles	SVM + Dutch sentiment lexicons and Pattern lexicon	Internet Movie (IMDB) dataset	Positive and negative
[115]	To highlight the emotions and polarity communicated by an article liable to increase the prediction regarding its acceptability by the audience	RF + NRC suite of lexica: EmoLex11	Medium (the articles on the online publishing platform)	Negative and positive, joy, sadness, anger, fear, trust, surprise, disgust and anticipation
[116]	To monitor transportation activities (accidents, vehicles, street conditions, traffic volume, etc.) To make a city-feature polarity map for travellers	Fuzzy ontology + SentiWordNet	Reviews from Twitter, Facebook and news	Positive, neutral or negative
[117]	To classify polarity of patient experiences of drugs using domain knowledge	Hybrid approach: FactNet, the knowledge base of polar facts	Drug reviews	Positive and negative
[118]	To use sentiment analysis and present a way to find relationships between tweets based on polarity and subjectivity	K-means algorithm + AFINN lexicon + TextBloB	Twitter data	Positive and negative
[119]	To propose a novel text representation model named Word2PLTS for short text sentiment analysis by introducing probabilistic linguistic terms sets (PLTSs) and relevant theory	SVM + SentiWordNet	Movie reviews (MR): Stanford Twitter Sentiment (STS): Tripadvisor reviews (TR)	Positive or negative
[120]	To compute the sentiments of social media posts	Fuzzy rule-based system + AFINN + VADER + SentiWordNet	Twitter datasets	Positive, negative or neutral
[121]	To extract user’s opinions and test them in two different datasets in English and Persian by introducing a part-of-speech graphical model	SVM + SentiWordNet,	Twitter, Iranian stock market	Positive or negative
[122]	To study Polarity Aggregation Model performance by extracting aspects of monument reviews and assigning to them the aggregated polarities	Deep Learning SAMs	Tripadvisor, English reviews	Positive, negative or neutral
[123]	To address the new methodology for dynamic modelling of customer preferences based on online customer reviews	Fuzzy + SentiWordNet	The online customer reviews of competitive hair dryers (Amazon.com)	Positive, neutral, and negative
[124]	To focus sentimental analysis on "times of India" movie review database	RF + SentiWordNet	Movie review dataset	Positive, negative and neutral

**Table 10:** Summary of hybrid approach (combination more than one of machine learning method with lexicon-based approach)

Reference	Objective	Method used in Hybrid Approach for Opinion mining	Materials	Output
[106]	To evaluate, analyse and classify the opinions on behalf of user tweets toward smart devices	NB + SVM + lexicon dictionary	Twitter tweets	Polarity: positive or negative and emotion: anger, joy, sadness, disgust, fear and surprise
[125]	To store, query and analyse streaming data	knowledge-based + machine-learning + 3-way classification process + SentiWordNet	Twitter dataset	Positive, negative and neutral
[126]	To examine the sentiment expression To classify the polarity of the movie review on a scale and perform feature extraction and ranking To train multi-label classifier to classify the movie review into its correct label	RF + DT + NB + k-NN + SentiWordNet	Rotten Tomatoes movie review dataset	Positive and negative
[127]	To provide an automatic and accurate polarity classification of Twitter messages	NB + SVM + DT (J48) + KNN + SentiWordNet	Twitter messages	Positive or negative
[128]	To study public emotions and opinions concerning the opening of new IKEA stores	EN + LR + NB + SVM + NN + RF + English sentiment dictionary	Twitter texts, IKEA-related topics	Positive and negative
[129]	To perform effective sentimental analysis and opinion mining of web reviews using various rule-based machine learning algorithms	DT + NB + SentiWordNet	Text reviews	Strong-positive, positive, weak-positive, neutral, weak-negative, negative and strong-negative
[130]	To shortlist words that help in sentiment cognition	Fuzzy entropy + k-means clustering, LSTM + SentiWordNet	Movie review datasets (IMDB)	Positive or negative
[103]	To employ an emotion detection technique for sentiment classification	NB + SVM + NNs, LogN, RF, CART + NRC emotion lexicon	Twitter	Positive, negative and neutral
[131]	To deploy the phrase level sentiment analysis to classify online reviews into positive and negative polarities	fuzzy entropy + k-means clustering + SentiWordNet lexicon	Movie review, Pang-Lee and the IMDB dataset	Positive and negative
[132]	To present a sentiment polarity detection approach that detects sentiment polarity of Bengali tweets	Multinomial NB + SMO(SVM)) + SentiWordNet + Indian sentiment lexicon	Bengali Tweets dataset	Positive, negative and neutral

**Fig. 12:**
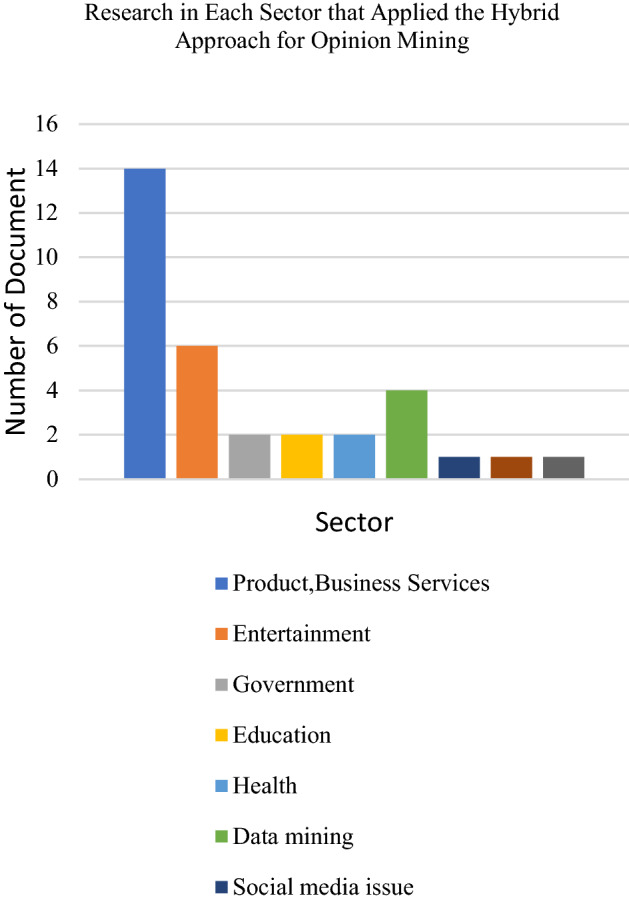
Chart of applications that used the hybrid approach for opinion mining

**Fig. 13:**
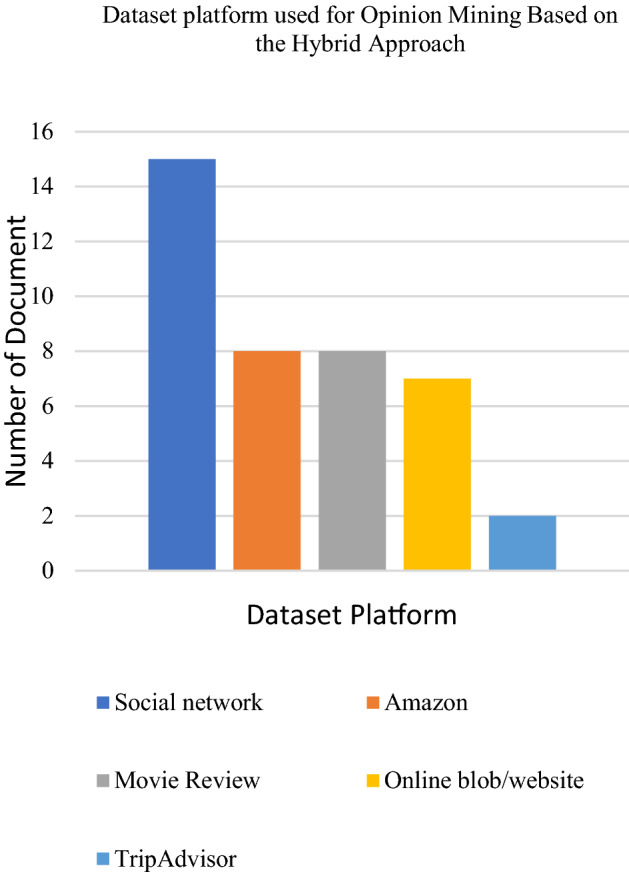
Chart of dataset platforms used in the hybrid approach for opinion mining

**Fig. 14:**
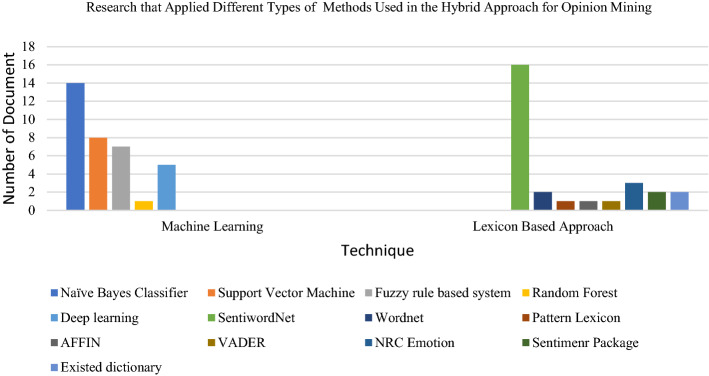
Chart of techniques used in the hybrid approach for opinion mining

The combination of the lexicon-based approach with machine learning is favourable to mine people’s opinions and emotions based on textual datasets according to specific research domains. Datasets from social media platforms such as Twitter and Facebook were seen as the most popular datasets used by researchers based on the reviewed papers. The IMDB movie review dataset comes next, followed by travel review datasets which have become well-known datasets to apply the hybrid approach. The following Figs. 12, 13 and 14 presents the distribution chart of articles according to application, technique and dataset platforms. The chart in Fig. 14 shows that NB is the most employed machine learning technique and SentiWordNet is one of the popular lexicon types used by the researcher. NB application in opinion predictions for various domains is due to its simplicity and fast processing time. The simple structure of this method makes it easy to implement and results in a high level of effectiveness. Meanwhile, SentiWordNet easy implementation in searching the opinions contributed to the frequent usage of the dictionary by the researchers. In addition, most of the researchers either use only one or more than one of the machine learning methods. For example, several researchers only employed NB or SVM and used a dictionary-based approach as the lexicon-based and the SentiWordNet and NRC emotion lexicon as the lexicon dictionary. Other than that, researchers combine more than one method of machine learning such as Naïve Bayes, Support Vector Machine, Decision Tree (J48) and the dictionary-based approach as their hybrid approach.

### Kansei approach

Recently, in the opinion mining-related domain, the Kansei approach was a new method implemented by the researcher. The Kansei approach has been used to study emotions toward certain entities based on textual data, such as product reviews. After reviewing papers that utilised the Kansei approach, we found that most research had focused on using emotions as the mechanism for measuring people’s expressions toward certain entities. It makes the Kansei approach one of the possible opinion mining approaches that can help in enhancing and improving techniques to mine people’s opinions. Among the existing Kansei approaches frequently used are Kansei Engineering (Type 1) and Kansei evaluation model techniques.

This research has used the Kansei approach to study visual content and investigate the evoked emotions in extremist YouTube videos among younger viewers [133].The method help in finding the specific emotion regarding content on the online social platform, but it does not involve finding any score of emotion that can help enhance the accuracy of the emotion classification. Different from this, researchers use the Kansei approach to construct the Kansei evaluation model for analysing product design from product reviews on the web by applying NLP methods based on the business/product domain [134]. From those methods, it can calculate and recognize the related scores evaluated by subjective experiments. The method is useful for products design that is highly had relation to people feeling. However, this method only focused on finding the product design-based people’s opinions according to reviews on online platforms.

Opinion mining using Kansei has not been fully explored yet, but recently, several articles have used the combination of the Kansei methodology with the text mining technique. Based on business/services domain application, Hsiao et al. [135] had used Kansei Engineering and text mining to analyse opinions regarding hotel services from people’s comments online review. Kansei Engineering, which is one of the methods in the Kansei approach, also uses emotions as the mechanism for evaluating people’s perceptions toward certain entities to mine people’s opinions based on text datasets. The hybrid approach between Kansei Engineering and text mining was effective in extracting and analysing the relationship between the consumer’s emotion and service characteristics that can help to improve the development of services and product for the hotel domain. However, this method had not involved any degree of values on the extracted emotion, and there had the participation of polarity classification. Recently, we can see the development of new research that integrated the Kansei approach and machine learning in mining people’s opinions. Research by Li et al. [136] was different because it combined Kansei Engineering and machine learning techniques such as Support Vector Machine (SVM) to analyse reviews of online stores from online shopping web pages and had involvement of degree words polarity classification. It was found that the integrated method helped in solving the opinion mining gap that only focused on the polarity classification of the positivity and negativity of the review texts and effectively assisted designers and manufacturers in recognised customers’ emotions to products design through inputting the review texts to facilitate the process of product design. Research of Hsiao et al. and Li et al. have become relevant foundations for the implication of the Kansei approach on another domain. For instance, the combination of the Kansei approach and machine learning technique for opinion mining in the national security domain is a matter that can be further explored. Table 11 presents the list of reviewed articles regarding the Kansei approach.

**Table 11:** Summary of papers reviewed using the Kansei approach for mining people’s opinions

Reference	Aim	Method	Material	Sector
[134]	To construct a Kansei evaluation model from product reviews on the web for product design by applying NLP methods to impressions	Kansei Evaluation model	Web review texts, Japan	Product design
[133]	To study visual content and investigate the evoked emotions in extremist YouTube videos among younger viewers	Kansei Engineering	YouTube videos	Extremist "Dark Side"
[135]	To develop guidelines for hotel services to help managers meet consumer needs	Kansei Engineering and Text mining	TripAdvisor review	Online hotel service
[136]	To extract and measure users’ affective responses toward products from online customer reviews	Kansei Engineering and machine learning	Online store reviews on the online store, the web pages of online shopping	E-commerce
[137]	To analyse the associations between service design elements (property space) of CBLS and customers’ Kansei perceptions	Kansei Engineering and Text mining	Google, Bing, Yahoo (CLBS keyword)	Hotel services (business)

### Drawbacks of opinion mining

Opinions and emotions from textual datasets, such as sentences from reviews, text in online news and blogs and whatever people post on social media, can be extracted using opinion mining techniques. However, the results extracted from opinion mining are in the form of sentiments or opinions, which are either positive, negative or neutral. Specific emotions of opinions, such as anger, sadness, etc., in the domain of national security, have not been fully explored in the opinion mining realm. Several researchers have been extracting emotions based on text. However, challenges exist when extracting emotions from text since more than one technique is needed, and this can require significant time. It must also involve a certain library that functions to look up the right emotion of the word. Some issues also exist when it comes to finding the best technique and method in classifying and extracting people’s opinions and emotions. Each opinion mining technique has its own difficulties and deficiencies. Opinion mining techniques that use machine learning and the lexicon-based approach do not assign identified emotions to specific domains. It would be helpful to mine people’s opinions within text according to specific domains.

Based on all research discussed in this study, Kansei Engineering has proven to be a potential method for evaluating the emotions of a certain entity. Overall, there is a gap to be addressed: combining Kansei Engineering with the opinion mining hybrid approach (the combination of machine learning techniques and lexicon-based approach) to extract and mine existing emotions and opinions within text in cyberspace according to specific domains, such as national security. Moreover, Kansei Engineering involves several steps to assess emotions towards a specimen. In preparing the assessment, there is a need a human involvement to collect a set of evaluation words suitable for evaluating the specimens in interest, arrange the evaluation word space, and choose suitable evaluation words to be used for the assessment. The collection of words from this approach can be utilised to develop a dictionary that can act as a lexicon in mining people’s opinions. It is similar to the existed lexicon such as the NRC emotion lexicon that had the same method in constructing their dictionary. The creation of the list of a word in the NRC emotion lexicon was based on human involvement in finding the word and evaluating the related emotion.

### Challenges for utilising machine learning, lexicon-based and Kansei approach in opinion mining

Researchers have been using opinion mining in business and product development sectors because it can help in mining people’s opinions regarding products. From these results, the product capability can be enhanced. Opinion mining is also used in government and health, and its application is still expanding. However, challenges exist in opinion mining applications such as the need for a dictionary that can be used in a different domain to produce a polarity score for a dataset. For example, Fischer and Steiger [72] have stated that regarding the health sector, limitations do exist on the use of dictionaries when conducting their research. Their problem was finding a specific dictionary for classifying medical literature. Other than that, when extracting emotions based on text, completing such a task is challenging due to the limitation of domain-specific emotion words. It depends on the existing library for scoring the opinions and emotions of words. Asghar et al. [138] realised that to extract the emotion based on the sentence, and there is a limitation on the ability to incorporate domain-specific words and automatic scoring of such words without performing a lookup operation in the existing library, such as SWN.

There is also a problem with the method used for mining people’s opinions and emotions. Although the Kansei approach has proven to be a method capable of determining people’s emotions regarding certain entities or artefacts, there have been several challenges that require further enhancements for this technique. Most researchers had adopted manual ways to combat this issue, such as making a questionnaire. Finding the right emotion by using this method requires significant time. For example, it has been stated that traditional SD questionnaires are widely used in the Kansei approach. This method is reliable but cumbersome because some research can take several years to complete, and hundreds of respondents must be involved [139]. This is challenging because Kansei is still a new approach and has limitations such as the lack of a systematic method for assigning scores to entities for emotion evaluation experiments in research. In 2018, Yamada et al. [134] implemented a text mining technique to perform Kansei evaluation for a product design. They found that the method is useful, and it is in automatic form. However, they had stated that some problems must be fixed such as the necessity to provide an appropriate score to entities used in the subjective evaluation experiment.

## Future research directions of opinion mining for national security

Future works should be based on the theoretical findings of the opinion mining method and the systematic literature review accomplished in this research. In our analysis, the results show that opinion mining had been utilised in several popular domains such as business, stock market and entertainment. In the articles surveyed in this SLR, most of the research has reported successful experiments using various techniques to mine people’s opinions based on text in cyberspace. Domain-specific emotion words are the limitation when extracting emotions based on text because of the high dependency on the existing library to determine opinions and emotions of words. Kansei approach has the potential to address the gap. These findings encouraged us to explore elevated techniques for opinion mining-related work in the domain of national security.

### National security overview

The end of World War II raised the term “national security” in American politics and held the attention of many throughout those years. The early development of national security had focused more on the military. Nowadays, the present concept covers a broad range of non-military aspects. To fit and adapt to the trending or current occurrences around the world, the concept of national security will continue to develop. National security is a category in political science [140]. It is a dynamic situation where the state and the society can be protected from threats of armed aggression, political dictatorship, and economic coercion. Two main concepts can define national security: to ensure the nation’s security and to secure the citizens [141].

When a country confronts direct and indirect threats, the government must mobilise its national security system [142]. National security refers to a country’s ability to be free from internally or externally threats to its core values. For example, social threats may include hostility from neighbouring nations, invasion of a terrorist group as well as global economic trends that have an impact on the country’s well-being. In distinct cases, dangers or threats may be considered a natural disaster or an outbreak of viral disease. Threats may affect the harmony and sovereignty of the country. Economic, political and social issues are of high interest and often debated in many nations since the elements of national security can be influenced by these issues. Military and non-military are the basic national security elements. Military security is the ability of a nation to secure the nation or intercept military violence from the outside. The non-military element is related to political security, food security, economic security, human security, energy and natural resources security, environmental security, border security, cybersecurity and health security [143]. Thus, an association between national security elements with citizens’ emotions must be studied so that efforts to maintain and strengthen these elements can be implemented [144].

### Hybrid approach of machine learning, lexicon-based and Kansei approaches for opinion mining in national security domain

Opinion mining is an emerging field of data mining that can be utilised to extract information, such as people’s opinions and emotions, from a vast volume of reviews and text on social platforms regarding any product or topic. Based on the reviewed articles, several methods have been used for opinion mining, such as the machine learning technique, the lexicon-based approach, the hybrid approach and the Kansei approach.

There are many drawbacks and difficulties that have been stated in various research regarding opinion mining techniques, such as lack of specific emotions in opinion mining research and the efficiency of machine learning techniques and lexicon-based approaches. Therefore, this research suggested to employs the Kansei approach that can be combined with machine learning technique and lexicon-based approach as a hybrid approach. However, the liability of the Kansei approach is the use of emotions and the evaluation process in determining the right and specific result of people’s emotions towards an artefact. Even though this method was not annotated with the polarity score, it can be solved by combining the Kansei approach with the machine learning technique and lexicon-based approach for the dictionary establishment for the national security domain. The machine learning technique and lexicon-based approach will help to calculate the text polarity score and enhance the accuracy of the opinion result. Therefore, this research presents a new domain: using the hybrid approach for opinion mining in national security.

Based on the review of the selected papers in the previous chapter, machine learning, lexicon-based approach and the Kansei approach demonstrated their capability of extracting people’s emotions in opinion mining. However, lack of domain-specific emotion words is the limitation faced when extracting emotions based on text due to high dependency on the existing library for scoring the opinions and emotions of words. The existing libraries that included emotions are NRC Word-Emotion Association Lexicon (known as NRC Emotion lexicon or EmoLex) and NRC Emotion Intensity Lexicon (called as Affect Intensity Lexicon). NRC Word-Emotion Association Lexicon is the emotion lexicon constructed for the English language, and it can classify text into eight categories of emotions and sentiment such as anger, anticipation, disgust, fear, joy, sadness, surprise and trust, positive and negative that different from the NRC Emotion Intensity Lexicon. The lexicon is not able to classify text into positive or negative sentiment because it contains the list of English words and their associations with only eight basic emotions (anger, anticipation, disgust, fear, joy, sadness, surprise, trust).

Thus, the Kansei approach can be utilised to complement this gap for the development of a dictionary that incorporates domain-specific words in a specific domain such as national security in opinion mining. For future research, this study suggests adopting a hybrid approach by combining the machine learning method and the lexicon-based approach with the Kansei approach to mine people’s opinions and emotions for national security. The emotions can be used as the parameter to relate with the national security risk using various scenarios such as anger and fear toward certain bad political issues that can bring unwanted risks such as riot, coup, terrorism, and civil war.

Machine learning and lexicon-based approach can classify and predict people’s opinions, while the Kansei approach can be used as a method to clarify people’s emotions in the national security domain. This hybrid approach will enable researchers, businesses and governments to apply the method to observe sentiments and emotions simultaneously for national security observation purposes. The expected output from this combination would be the evaluation of people’s sentiments and emotions with the inclusion of the score value of polarity according to the national security element.

### Benefits of performing opinion mining in national security

Various activities in cyberspace pose a risk to national security, such as cyber rumours, fake news websites and hate speech [145]. These types of threats in cyberspace can be significant risks to national security [146]. Individuals involved in such activities can indirectly become conspirators since every cyberspace user has a distinct persona, opinion, religion and emotion. They can willingly or unwillingly believe these false rumours and continue to endorse and share them with others. These types of human emotions and behaviours can affect cyberspace. Thus, emotion is deemed a crucial mechanism to detect threats towards national security. Since cyberspace has an emotionally rich nuance and space where people can express their emotions, sentiments and opinions, the connection between emotion and hate speech in cyberspace is undeniable [147]. Related research on emotion in the national security field had found that fear and anger affect politics, which is one element of national security [148]. The relation between emotion and national security elements can be seen in how humans react towards issues related to environmental security. A study did find that ‘hope’ is a reaction that people have towards climate change [149].

The implementation of opinion mining in the national security domain is crucially beneficial. The reason is that most information in the online system is displayed in textual form. A substantial amount of textual data can be generated since it is usual for an individual or persona in cyberspace to express emotions through words or text [150]. By utilising opinion mining in detecting threats in cyberspace, the state of national security can be strengthened.

## Limitation

This research intends to incorporate all published literature, such as articles, press articles, and research papers, referring to the implementation and application of opinion mining techniques in cyberspace, including the utilisation of the Kansei approach. It uses a systematic literature search methodology to collect valuable information from a collection of available literature. It reveals current developments of opinion mining and the Kansei approach in mining people’s sentiment, paving the road forward for further research. The scope of this work is restricted to the technique of opinion mining and the Kansei approach in mining people’s sentiments based on text to implement in the national security domain. Since 2003, research in this field has been growing and continues at a steady pace of development.

## Conclusion

Opinion mining has been a helpful mechanism in finding people’s sentiments and emotions based on text in cyberspace. Based on our research findings, in most of the reviewed papers in this research, various domains do exist that usually employ opinion mining, such as business/products, transportation, health, government, entertainment, and education. It shows the involvement of opinion mining capabilities in various domains. However, there are several drawbacks from the implication of opinion mining techniques that have been discussed in this research. Thus, this study can help as a reference for future research on finding and determining the suitable method for future new research domains such as national security that was suggested. Although mining people’s opinions and emotions for national security is relatively new research, it should be explored and investigated by researchers to enhance the literature within the national security field. This will further secure and strengthen a state’s national security from unwanted threats. This research suggests that the combination of the machine learning method, lexicon-based approach and the Kansei approach can be a possible mechanism for evaluating people’s emotions within the text. This includes the text’s opinion polarity and possible emotions flag that can influence people’s acceptance of information in cyberspace.

## Data Availability

All papers studied in this systematic review are available in SCOPUS, IEEE Xplore, ACM Digital Library, SPRINGERLINK and ScienceDirect. Please see the references below.

## Abbreviations

ACM: Association for Computing Machinery
IEEE: Advancing Technology for Humanity
NLP: Natural Language Processing
COVID-19: Coronavirus disease 2019
KE: Kansei Engineering
LSTM: Long short-term memory
LR: Logistic Regression
NB: Naïve Bayes
SVM: Support Vector Machines
DT: Decision Tree
SGD: Stochastic Gradient Descent
NNs: Neural Network
RF: Random Forest
LDA: Latent Dirichlet Allocation
KNN: K-Nearest Neighbour
ML-KNN: Multilabel K-Nearest Neighbours
ME: Maximum Entropy
CRF: Conditional Random Fields
AdaBoost: Adaptive Boosting
BFTree: Best-First Decision Tree
OneR: One Rule
CNN: Convolutional Neural Network
ANN: Artificial Neural Network
DBN: Deep Belief Network
DNN: Deep Neural Network
RNN: Recurrent Neural Network
GRU: Gated Recurrent Unit
BERT: Bidirectional Encoder Representations from Transformers
BPNN: Back-Propagation Neural Networks
SD: Semantic differential
IMDB: Internet Movie Database
PMI: Point-wise mutual information
MPQA: Multi-Perspective Question Answering
SWN: SentiWordNet
Vader: Valence Aware Dictionary and Sentiment Reasoner
